# Disruptions to essential health services in Kenya during the COVID-19 pandemic – February 2020–May 2021

**DOI:** 10.1017/ash.2022.151

**Published:** 2022-05-16

**Authors:** Matthew Hudson, Carolyn Herzig, Godfrey Woelk, Evelyn Wesangula, Rhoderick Machekano, Rose Masaba, Benjamin Park, Elizabeth Bancroft

## Abstract

**Background:** The COVID-19 pandemic disrupted essential health services (EHS) delivery worldwide; however, there are limited data for healthcare facility (HCF)–level EHS disruptions in low- and middle-income countries. We surveyed HCFs in 3 counties in Kenya to understand the extent of and reasons for EHS disruptions occurring during February 2020–May 2021. **Methods:** We included 3 counties in Kenya with high burden of COVID-19 at the time of study initiation. Stratified sampling of HCFs occurred by HCF level. HCF administrators were interviewed to collect information on types of EHS disruptions that occurred and reasons for disruptions, including those related to infection prevention and control (IPC). Analyses included descriptive statistics with proportions for categorical variables and median with interquartile range (IQR) for continuous variables. **Results:** In total, 59 HCFs in Kenya provided complete data. All 59 HCFs (100%) reported EHS disruptions due to COVID-19. Among all HCFs, limiting patient volumes was the most common disruption reported (97%), while 56% of HCFs reduced staffing of EHS and 52% suspended EHS. Median duration of disruptions ranged from 7 weeks (IQR, 0–15) for inpatient ward closures to 25 weeks (IQR, 14–37) for limiting patient volumes accessing EHS. Among HCFs that reported disruptions, the most cited reason (ie, 95% of HCFs) was fewer patients receiving services. The most common IPC-related reason for disruption was diversion of resources to accommodate physical distancing measures (76%) followed by COVID-19 outbreaks among patients or staff (34%); staff shortages due to COVID-19 illness (25%) or perceived infection risk (19%); and lack of adequate personal protective equipment (20%). **Conclusions:** Most HCFs reported disruptions to EHS during the pandemic, including many that were related to IPC. Some disruptions may be mitigated by strengthening IPC infrastructure and practices, including protecting healthcare personnel to prevent staffing shortages.

**Funding:** None

**Disclosures:** None

